# You Can't Be With Your Patients All the Time—Patient and Staff Views of a Wearable Vital Signs Monitoring System

**DOI:** 10.1111/jan.70610

**Published:** 2026-04-20

**Authors:** Clare Edwards, Holly Edmundson, Elizabeth King, Cristian Roman, Peter Watkinson, Sarah Vollam

**Affiliations:** ^1^ Nuffield Department of Clinical Neurosciences University of Oxford Oxford UK; ^2^ Oxford NIHR Biomedical Research Centre Oxford UK; ^3^ Nuffield Department of Primary Care Health Sciences University of Oxford Oxford UK; ^4^ Oxford Allied Health Professions Research and Innovation Unit Oxford University Hospitals NHS Foundation Trust Oxford UK; ^5^ Institute of Biomedical Engineering, Department of Engineering Science University of Oxford Oxford UK; ^6^ Oxford Critical Care, John Radcliffe Hospital Oxford UK

## Abstract

**Aim:**

To explore staff and patient perception of the newly co‐developed wearable monitoring system (WMS), including acceptability of use in clinical practice.

**Design:**

Pragmatic qualitative descriptive study.

**Methods:**

Semi‐structured interviews were conducted with 12 patient participants and eight staff members between June 2023 and August 2024, and were analysed thematically.

**Results:**

Three themes were identified, building on previous qualitative work around the use of WMS on hospital wards. The first theme—*centralised continuous monitoring enhances care—*explores how WMS provides staff with a means to provide safe, efficient care with the ability to see the vital signs away from the patient. Patients reported feeling safer, knowing they were being monitored when staff were not at the bedside. The second theme—*human connection at the bedside*—considers how both patients and staff emphasised that the system should not replace nurse/patient interactions and face‐to‐face care, even though it provided patients with a stronger sense of independence. The final theme—*system usability and integration into care—focuses* on use of the system in clinical practice and implications for the future.

**Conclusion:**

Wearable monitoring systems have the potential to support nurses to provide safer, more efficient care, whilst providing reassurance to patients. However, centralised monitoring should not replace face‐to‐face clinical contact, and careful consideration should be given to who would benefit most from the technology.

**Impact:**

This study extends existing knowledge of the impact of WMS from being a tool to enhance patient safety to an intervention to improve nurse efficiency and patient experience, within the context of a high‐demand surgical ward.

**Patient and Public Contribution:**

Patients and members of the public were involved in study design and data collection. Their contributions included participating in advisory groups, ensuring the research addressed patient‐relevant priorities.

## 1 Introduction

Surgical patients are at high risk of clinical deterioration, usually preceded by changes in vital signs (Khanna et al. 2025). To aid prompt identification of clinical deterioration, nurses regularly measure patients' vital signs, with the time between measurements based on how close to normal values each measurement is. This is often supported by scoring systems such as the UK National Early Warning System (NEWS), which protocolise the frequency of vital signs measurements (England 2017). However, this risks delays in detecting changes that occur between these measurements and could have been acted on more quickly (Khanna et al. 2025). In intensive care units (ICUs), vital signs are usually measured continuously using wired monitoring systems. Although wired monitors give rapid warning of changes in vital signs, which may indicate a clinical deterioration, these systems are often uncomfortable for the patient and restrict movement (Areia, Biggs, et al. 2021).

## 2 Background

To address the limitation of intermittent vital signs measurements, wearable monitoring systems (WMS) using small, wireless wearable devices are increasingly being used on surgical wards internationally (Bignami et al. 2025; Khanna et al. 2025). These WMS offer continuous measurements of vital signs, including heart rate, breathing rate and blood oxygen levels, but are comfortable to wear and do not restrict patient mobilisation, which is important for recovery after surgery (Kokotovic et al. 2021). There is increasing evidence that WMS improve patient outcomes in surgical populations, such as mortality and ICU readmission, although it is currently limited and based on small‐scale studies and feasibility trials (Areia, Biggs, et al. 2021; Khanna et al. 2025).

To ensure WMS can be successfully implemented into practice and maximise patient benefit, it is important to understand the experiences of patients and staff using these systems. However, the increasing use of WMS data and exploration of acceptability remains limited (Sprogis et al. 2019). Previous qualitative studies have separately assessed staff and patient perceptions of different WMS systems (Buss et al. 2024; Downey et al. 2018; Kooij et al. 2022; Leenen et al. 2022), with one previous study exploring the use from both perspectives (van Noort et al. 2024). All studies identified a perception that WMS improved patient safety by allowing nurses access to continuous vital signs data. However, exploration of the mechanism for supporting patient safety and consideration of the impact on nursing practice and workflow from both patient and staff perspectives is limited and uncertain. There is some evidence to suggest WMS may support nursing workload prioritisation in pandemic conditions (Buss et al. 2024). However, although such systems may support nurse decision‐making (Leenen et al. 2022), one study suggested nurses found WMS increased their workload (Kooij et al. 2022) and a further study found disagreement between respondents regarding whether workload was increased or decreased during system use (Weenk et al. 2020). The impact of WMS on nurse workload, and the mechanism for the impact, are therefore unknown.

### 2.1 Context

This qualitative study was part of a single‐site feasibility randomised controlled trial (RCT) of a new WMS in a hospitalised surgical population (ClinicalTrials.gov ID NCT05118477). The WMS comprised wearable devices (an adhesive chest patch and wrist‐worn pulse oximeter) that connect via Bluetooth to bedside tablets that relay vital‐sign information to the central nurse station monitor via Wi‐Fi. The system was developed in multiple stages following wearability, accuracy and acceptability testing (Areia et al. 2020; Santos et al. 2021). Data were viewable on a central screen at the nurses’ station via a user interface co‐developed with the ward nursing staff prior to the study. Further details of the system are reported elsewhere (Buss et al. 2024; Santos et al. 2021). For this trial, participants were randomised into two groups. In the intervention group, data from the WMS were visible to both patients and nursing staff at the bedside and at the central nurses’ station. Data from the control group were blinded. The system generated audible alerts based on NEWS2 (England 2017) thresholds from the wearable monitoring data at the nurses’ central station for the intervention group only. Routine care, including regular observation rounds, continued whilst patients wore the WMS devices. This study ran concurrently with the RCT to explore user perceptions of the intervention.

### 2.2 Study Aim

To explore staff and patient perception of a newly co‐developed WMS, including acceptability of use in clinical practice.

## 3 Methods

The COREQ (Consolidated Criteria for Reporting Qualitative Research) checklist was used to prepare this manuscript (Tong et al. 2007).

### 3.1 Study Design

This study was part of a wider mixed‐methods project, comprising a concurrent RCT and qualitative exploration of the trialled intervention. We undertook an exploratory qualitative descriptive interview study with patients and staff who had used the system. We followed a pragmatic approach, suited to the use of mixed methods research where the aim is to understand user perspectives of the intervention being trialled to inform future practice (Creswell and Plano Clark 2011; Doyle et al. 2019). This approach allowed us to explore the implementation and impact of the intervention from different user perspectives, and to understand what works for them. Data were collected between June 2023 and August 2024. Semi‐structured, one‐to‐one interviews following a topic guide allowed flexibility to respond to participant comments and exploration of emerging insights.

### 3.2 Setting and Recruitment

The study took place on a 19‐bed general Surgical Emergency Unit (SEU) in a large tertiary university teaching hospital in central England. All patients who were enrolled in the RCT were invited to take part in the interviews at the point of initial consent. If they expressed an interest in being interviewed, the participant was provided with a separate Patient Information Sheet (PIS) describing the interview process. Staff members working on the ward who cared for patients wearing the WMS were approached, aiming for maximum variability in terms of professions, grades and experience of the system. If they expressed an interest in being interviewed, they were provided with a PIS describing the interview process and given the contact details of the research team to confirm if they wished to participate.

### 3.3 Inclusion and Exclusion

All patients enrolled in the RCT were eligible to participate. Patients were eligible for the RCT if they were surgical patients over the age of 18, admitted to the ward, able to speak and understand English, and able to provide written consent. Patients were required to be clinically stable before entering the study. This was defined as having a NEWS2 score of 2 or less for the previous 6 h, not being monitored continuously at the bedside (using a Philips Cardiac monitor (Healthcare PP 2025), based on clinical acuity), and nurses were monitoring their vital signs observations at no more than four‐hourly intervals. Patients were ineligible if they were unable to wear the monitoring devices (e.g., they had an intracardiac device or allergy to adhesive dressings) or were anticipated to be discharged from the ward within 24 h. All ward staff with experience of caring for patients wearing the WMS were eligible to participate.

### 3.4 Data Collection

Patient interviews were conducted during their hospital stay—face‐to‐face in a side room or at the bedside, or after hospital discharge, either by telephone or in person in a quiet room at a research facility based in the hospital.

All staff interviews were held in a room away from the ward area, or by video calling using Microsoft Teams or by telephone, depending on participant preference. Semi‐structured interviews were undertaken using topic guides (see supporting material for included questions). This was written collaboratively by the research team using the available literature and drawing on previous studies developing the WMS. This allowed for key questions to be explored whilst allowing participants to express their own individual experience of the system. We piloted the topic guide during the first staff and patient interviews and discussed this within the team. No substantial changes were required following piloting. Each interview was audio recorded and transcribed verbatim by a member of the research team.

### 3.5 Data Analysis

Data were analysed using the six steps of thematic analysis (Clarke and Braun 2013). In step one, two researchers (CE and HE), both research nurses working on the RCT with training in qualitative research methods, familiarised themselves with the initial data by reading through the interview transcripts. In step two, preliminary codes were generated in four initial transcripts, using NVivo 12 Plus software. These preliminary codes highlighted key elements of the data relevant to the research question. This commenced soon after the first few interviews were conducted and allowed for an iterative approach to the topic guide. Preliminary codes were discussed within the team (CE, HE, EK and SV, the two research nurses and two experienced qualitative researchers with qualitative methods training) in relation to the research question. Following the team discussion, coding of all transcripts was completed. Analysis continued through steps three and four. Initial themes were identified by grouping codes into categories, or sub‐themes, and then into broader themes. In step four, data from initial themes were collated and discussed, with refinements made using mind maps which explored links between codes and themes with all four qualitative researchers (CE, HE, EK and SV) during multiple research meetings. Any disagreements during the analysis process were resolved through discussion. Once the final themes were agreed (step five), the findings were developed into a narrative account of the themes with illustrative quotes (step six), where further analysis and refinement resulted in the findings presented below.

### 3.6 Sample Size

We used the concept of information power to estimate an adequate a priori sample size (Malterud et al. 2016). It was anticipated that up to 30 participants may be required to meet the aim of the study, given our relatively focused research question and two relatively homogenous participant groups, which suggested a comparably small sample size was likely to be sufficient. To assess the adequacy of our estimated sample size, we also considered the concept of data saturation as data collection progressed. This aligned with our pragmatic approach and allowed us to identify when sufficient data had been collected to answer our research question (Roberts et al. 2026). Recruitment ceased when no new codes were being identified, and it had been agreed as a team that data saturation had been achieved.

### 3.7 Ethical Considerations

The study was approved by the Wales Research Ethics Committee (REC) on 24 August 2021 (REC reference (Roberts et al. 2026)/WA/0250).

Researchers provided potential participants with written information about the study and ensured they were aware that participation was voluntary. All participants provided written consent and were reminded that they were free to withdraw from the study at any point until their data were anonymised. Although researchers did not anticipate that the interview process would cause distress amongst participants, their experience with qualitative interviewing enabled them to provide appropriate referrals to support services should the situation arise.

### 3.8 Rigour and Reflexivity

Rigour and reflexivity were considered using the four main concepts of trustworthiness: credibility, transferability, dependability and confirmability (Lincoln and Guba 1985). Credibility was ensured through CE's sustained engagement with the data during data collection and analysis, as well as through regular team discussions about reflexivity as the analysis progressed. The interviewers consisted of three experienced clinical research staff, two of whom recruited participants to the RCT and were therefore known to some of the patients and staff they interviewed. Their familiarity with the participants shaped the interview dynamics. This was acknowledged and addressed by engaging in critical reflection during the interview process, revisiting and appraising the data to evaluate if their own subjectivity influenced the interviewing process. The researchers used reflexive memos to record general impressions on the content and outside influences that may have affected the interview, such as time pressures and ward workload, which were considered during analysis. The inclusion of participant demographics as well as a detailed description of the setting and trial intervention supported transferability, allowing readers to make comparisons with their own clinical setting. The interpretation of the data was overseen by an experienced qualitative researcher (SV) within the research group who provided guidance on reflexivity throughout the process, which enhanced dependability. In addition, research diaries were maintained throughout the analysis, documenting key analytical decisions. Confirmability was achieved by using direct quotes to illustrate findings and the use of reflexive memos.

## 4 Findings

Interviews were conducted with 12 patients (six during their hospital stay and six after discharge) and eight staff members. Participant demographics are outlined in tables one and two. The ages of interviewed patients ranged from 18 to 81 years (median 49.5) (Table 1), and the ward length of stay (LoS) varied from 3 to 46 days (median = 16). The staff participants' experience ranged from newly qualified to more than 10 years (median = 3.5). Six staff members were registered nurses and two were Clinical Support Workers (CSWs), both of whom engaged daily with the WMS (Table 2). A high turnover in CSW staffing meant that only two permanent members of staff were available for interview.

**TABLE 1:** Patient interview demographic details.

Interview number	Gender	Age
P1	Female	81
P2	Male	21
P3	Male	53
P4	Male	65
P5	Male	73
P6	Female	38
P7	Male	76
P8	Female	59
P9	Male	51
P10	Female	53
P11	Male	29
P12	Female	18

**TABLE 2:** Staff interview demographic details.

	Gender	Band	Years qualified
S1	Female	6—Staff Nurse	4
S2	Female	6—Staff Nurse	3
S3	Female	7—Staff Nurse	5
S4	Female	5—Staff Nurse	< 1
S5	Female	6—Staff Nurse	5
S6	Female	3—CSW	2
S7	Male	3—CSW	> 10
S8	Female	5—Staff Nurse	1

Three main themes were identified from the data: The first theme, *Centralised Monitoring Enhances Care*, explores how WMS offered staff the means to provide safe and efficient care, identifying the value of viewing trends in vital signs away from the patient, whilst patients felt safer knowing they were being monitored even when staff were not at the bedside. The second theme, *Human Connection at the Bedside*, describes how, despite the benefits to workload and patient safety, both patients and staff emphasised that the system should not replace nurse/patient interactions and face‐to‐face care. The final theme, *System Usability and Integration into Care*, focuses on system design and usability, how it may be integrated into clinical care, and implications for the future.

In the following sections, these themes and their subthemes are explored, alongside quotes from nursing and support staff (S) and patient (P) participants, demonstrating both shared and divergent perspectives within the themes.

## 5 Theme 1: Centralised Monitoring Enhances Care

This theme explores the ways in which the use of the WMS was perceived to improve or enhance nursing care. This concept of WMS supporting patient safety has been identified previously (Areia, Biggs, et al. 2021; Buss et al. 2024; Leenen et al. 2022), but in this theme, we build on these earlier findings in more detail and with contrasting patient and staff perspectives. We also extend our understanding further by exploring the impact on nurse workload. These concepts are explored in the following four sub‐themes: (1) early recognition of the deteriorating patient; (2) prioritising care from the nurses station; (3) vital signs trend supports better care planning and (4) supporting comfort, rest and physical autonomy.

### 5.1 Sub‐Theme 1.1: Early Recognition of the Deteriorating Patient

Similar to previous studies, interviewed staff felt that the system was useful for early recognition of patient deterioration. In this study, nursing staff evaluated different patient cohorts, suggesting the WMS was particularly beneficial for managing patients who were more acutely unwell. This included recently post‐surgical patients, severely unwell patients, or those undergoing treatment changes (such as weaning from oxygen), all considered periods of higher risk of deterioration. Nurses perceived the WMS as enabling them to observe trends in vital signs when they were away from the bedside.

S3: ‘One of the patients we've had today; we've weaned him down off his oxygen and so it's been really helpful to be able to track what's going on without having to be at his bedside’.

Patient participants who would have fallen into the higher‐risk groups identified by the interviewed nurses described feeling aware of their vulnerability during that time. Whilst they stressed that the WMS should not replace face‐to‐face contact (which will be explored in the next theme), they also recognised that nurses couldn't be everywhere at once. Both nursing staff and patients felt reassured knowing that the system would alert staff to significant changes in condition. Several participants described specific cases when the alerting system had allowed for prompt actions that prevented deterioration or harm to the patient and aided an improved recovery path.

P11: ‘…my blood oxygen was low…. having the oxygen on immediately really helped… that was as a direct consequence of the band [the sats monitor]’.

S6: ‘it's just good at picking up earlier signs of deterioration. that patient who was getting out of bed, it started alarming, and no one was in the bay… we went to go and see him and probably saved him from having a fall’.

The consensus from both groups was that the system was unnecessary for less acutely unwell patients, such as those approaching discharge. This was described as a time when the aim was to decrease medicalisation and reduce interventions to promote independence. Conversely, one patient suggested that the WMS might have allowed him more time off the ward, facilitating an increase in independence, as he did not need to consider being around when nurses came to manually check his vital signs.

S1: ‘It's not quite so appropriate for patients who are surgically fit*…* they're free to go home but waiting for something social*…* they still feel very medicalised if they've still got all the equipment on whereas we try and promote independence’.

P3: ‘…for the last few days it would have had a different role*…* I would have been learning that independence and not have to worry about whether or not I'd gone off for a walk [off the ward]…every time they came to do my readings’.

In summary, both patients and staff discussed the benefit of the WMS in promoting patient safety through the detection of clinical deterioration when used in conjunction with face‐to‐face rounds. Examples of where this benefit was seen in practice included acute and emerging situations, which were rectified quickly because of actions taken following the alerts at the central nurses' station. Both groups generally agreed that the WMS was only of benefit to those at risk of deterioration and should be removed as patients approach hospital discharge.

### 5.2 Sub‐Theme 1.2: Prioritising Care From the Nurses Station

Several nurses described the WMS as a valuable support tool that helped them monitor multiple patients simultaneously at the nurses' station. They reported that the system gave them confidence in prioritising their workload by deciding when they needed to focus on a particular patient and when they could attend to other tasks. They discussed feeling more able to provide continuity to all those in their care, having good oversight of their daily caseload and knowing that any signs of patient deterioration between face‐to‐face checks would trigger alerts.

S1: ‘I think it helps with workload. I'll do this quickly or that quickly and you think you can slot things in a bit quicker when actually if you can see those numbers, it's no I can't, and that needs to go to the top of my list, and I need to go there now’.

P5: ‘Well I think as far as I was aware it enabled people to keep a close eye on me without having to come into the single room*…* it frees up staff to see the patients who really need them*…* It was reassuring really*…*’.

In previous studies, both staff and patients have emphasised that WMS should not replace nurse–patient contact (Areia, King, et al. 2021; Buss et al. 2024). Whilst both groups in this study agreed that regular contact was important, most felt this did not need to include vital signs monitoring and that it would be quicker to use the WMS data. In support of this, both staff and patients described practical challenges faced by nurses completing and documenting routine observation rounds, specifically in locating working equipment and having to take it from bed to bed.

S1: ‘*…* where you've got six patients to look after and they're all needing things, and you've got [a patient needing] fifteen‐minute obs on top. It's easier to go right I can see my patient [on WMS], he looks fine and jot down the obs*…* We never have enough computers; we never have enough space’.

P6: ‘they would be able to just put their head round the curtain and just say everything's fine and that saves five or ten minutes of coming up with the trolley, oh no the ear thing doesn't work, the thermometer doesn't work, the cuff doesn't work and the machine the battery's gone and that happened lots’.

Several nursing staff discussed the logistical challenges of caring for multiple patients connected to the traditional wired monitors, which only displayed data and alerts at the bedside. Having to go to each patient's bedspace to see the monitor outputs was described as a time‐consuming and inefficient way of providing safe patient care.

S3: ‘Last week I had four on cardiac monitors all at the same time and so trying to get round to all of them at once was quite a challenge. Its fine if they were all in a bay together but mine were in two separate bays’.

Working on a busy surgical ward demands that the workload is continually reassessed to ensure patient care is appropriately prioritised (Aaberg et al. 2021). Clinicians using WMS reported that they were better able to see the ‘big picture’ of patients' health status on the ward and prioritise their workload whilst keeping their patients safe. The system was regarded as ensuring prioritisation of care for those who needed it most.

### 5.3 Sub‐Theme 1.3 Vital Sign Trends Support Better Care Planning

Patients described feeling that continuous vital signs data would contribute to more personalised care planning by offering a comprehensive picture of their current recovery progress rather than snapshots captured during the intermittent routine observations.

P6: ‘…rather than relying on something every four hours which can be misconstrued if that patient got up to go to the toilet five minutes before so their blood pressure might be higher or they might have been lying down for a long period of time and I think you would be getting a more balanced view of that patient's vital signs in my non‐medical opinion’.

Nurses described looking at trends in the vital signs, which can be viewed at the central station, and using these to support care planning discussions with members of the multidisciplinary team on the ward.

S3: ‘…i was doing something at the nurse's station and they [physiotherapist] came to talk to me and I said I can show you how he's looking right now’.

Patients felt that continuous monitoring provided a comprehensive, more balanced view of their vital signs than intermittent snapshots, facilitating more accurate treatment strategies. Whilst staff primarily valued the system for detection of deterioration and task prioritisation, some also used it for trend analysis and multidisciplinary team discussions to support clinical decision‐making.

### 5.4 Sub‐Theme 1.4 Supporting Comfort, Rest and Physical Autonomy

Quality sleep and uninterrupted rest are critical for post‐surgical recovery, influencing pain management, immune function and wound healing (Beswick et al. 2023). Patients interviewed commonly described having their sleep or rest disturbed by nurses undertaking vital signs measurements and by the alarms produced by the bedside monitors. Both nursing staff and patients felt that WMS could facilitate monitoring whilst creating a more restful ward environment. In addition, some nurses felt that avoiding nighttime disruptions not only supported better sleep but also helped maintain stable vital signs.

P6: ‘The big machine [wired monitor]. I mean that's a pain, isn't it? With the beeping. And yes to be able to do without that is a winner*…* If you're in that situation*…* you're feeling pretty grim so to have that [wired monitor] beside you, and then you've got other people who've got it as well, and so it's not just your machine, there's someone else's going off and alarms all the time. And then something falls off and it bleeps at you and there's somebody rushing around, sticking it back on you, pressing a button’.

S7: ‘I think this [WMS] monitoring is better for patients, so you don't need to wake them up in the middle of the night. Just waking them up can cause their BP [blood pressure] to go up or their heart rate’.

When discussing device comfort, interviewers explored patients' experiences with WMS devices in comparison to traditional monitoring equipment. The chest patch was consistently well received, with several patients noting it was so comfortable they often forgot they were wearing it. However, some patients reported minor discomfort, including skin irritation, pinching from the adhesive, or discomfort during removal—particularly amongst those with hairy chests. The pulse oximeter, which was worn around the wrist and had a wire connecting to a finger probe, was mostly comfortable, though some found it heavy or that their finger became sweaty with prolonged wear. Some patients also described the pulse oximeter as restricting hand movements due to the device and offered suggestions for potential improvements. Additionally, unlike the water‐resistant chest patch, the oxygen saturation monitor had to be removed when using the bathroom, which was also seen as an inconvenience and obstructive to their independence.

P4: ‘I found it [chest patch] no inconvenience whatsoever*…* There have not been any complaints and I even had a shower… It wasn't until I came home that I realised I still had the thing on my chest, so yes you become oblivious to wearing it*…* I did say at the time the one on my finger if that could be designed a little bit more thinner, like a thimble almost that would be a lot easier but I soon got used to it and it didn't bother me at all’.

P8: ‘I tried to go for a shower and trying to get everything [sats monitor] off, get dry, and put it back on again*…* I mean I took it off in the end. It just got on my nerves*…* That [chest patch] was alright but it started to pinch my skin a little bit as the glue was drying and it was catching me and I wasn't in a very good mood I'll tell you that now’.

Overall, the WMS was perceived to support the promotion of patient autonomy and independence. Both staff and patients highlighted the advantages of wireless monitoring, noting that it enabled patients to mobilise whilst still being monitored. Additionally, the compact bedside display used with WMS (a 10‐in. computer tablet) freed up floor space compared to the bulkier traditional wired monitor, further facilitating patient movement. One participant reported being unable to access his wheelchair until the wired monitor was replaced with the WMS device.

S2: ‘Even though they [patients] are in pain, they wanted to go to toilet [not use a commode whilst attached to a bedside monitor]. they want to maintain their independence*…* with the monitoring system, okay, that's fine—they can still be monitored because we're still looking at the screen’.

P10: ‘It [WMS] takes up less room which is a big bonus. The one I had in here [Philips] actually blocked off the doors in the end and I couldn't get to my wheelchair because it's such a big monitor’.

When contrasting the WMS with traditional wired bedside monitors, patients felt that bedside monitors disrupted sleep due to noise and frequent disturbances, whilst WMS offered a quieter, more restful environment. Whilst staff valued WMS for reducing nighttime disruptions and maintaining stable vital signs, both patients and staff highlighted its role in promoting mobility and autonomy through wireless monitoring.

## 6 Theme 2: Human Connection at the Bedside

Building on the findings of the previous theme and findings of previous studies, this theme explores the importance of maintaining personal interactions between patients and healthcare staff, which is important for reassurance, and how to balance this with the use of WMS to manage workloads.

In common with previous studies (Areia, Biggs, et al. 2021; Buss et al. 2024), interviewed nurses highlighted that direct patient interaction provides context to vital signs and an opportunity to gain a holistic overview of the patient's status, such as identifying non‐clinical causes for abnormalities. These concerns highlighted the importance of balancing technological efficiency with face‐to‐face care to ensure comprehensive assessments.

S5: ‘We still need to see each patient because you can tell so much from what the patients says to you or looks like to you so this system can't replace that’.

The importance of maintaining frequent patient‐staff interactions was discussed by many of the participants to assess physical and emotional well‐being. Patients reported that they valued being checked in on, appreciated opportunities to ask questions, and felt it facilitated structure to their day.

P3: ‘… there's something quite reassuring about someone doing your observations and talking to you and you know that the day is passing and its progressing and you can ask a question that might not be urgent but you know that someone will be around at that point’.

Several nurses and patients reported that having the bedside display of their vital signs improved patients' anxiety levels during what can be a very stressful time. They described this as offering the opportunity for patients to engage with their care, opening dialogue with their caregiver by providing additional information about their condition, which they valued. One nurse described an instance where the bedside display worried the patient, but they were easily reassured, and it was not seen as problematic.

S3: ‘I think actually some of them are quite anxious. So the gentleman that we've got at the minute has got mental health conditions and he's actually been reassured by having it, even when his saturations weren't as good because he could track it, he could see how he was doing and that gave him some control of the situation’.

P6: ‘I would much rather know all of that information and know how I'm progressing, whether that's positive or negative so that I know what we're going to do about it’.

There were differences in opinion about the suitability of WMS for confused or agitated patients. Some nurses reported feeling that the WMS may not be well tolerated in this patient cohort.

S4: ‘We often get people with cognitive impairment or who are agitated so it would be much easier if they didn't have to be hooked up to a machine like they are at the moment’.

Whilst WMS was generally well‐received, its perceived suitability for anxious or cognitively impaired patients varied—some found reassurance in viewing their vital signs, whilst others may have preferred the current intermittent monitoring where vital signs are not displayed to the patient. Both groups reported frequent patient‐staff interactions in addition to continuous monitoring, which remained an important source of comfort and support, as well as crucial to interpreting clinical conditions.

## 7 Theme 3: System Usability and Integration into Clinical Care

This theme examines how staff and patients perceived the system's design, usability and functionality, including its ease of integration into clinical workflows and its impact on patient care.

In common with a previous study of this system (Areia, Biggs, et al. 2021; Buss et al. 2024), staff perceived the WMS as straightforward, intuitive and easy to use. Suggestions for improvements included a larger screen at the nurses' station and more control to tailor alarms to individual patients. The bedside display (visible for the intervention cohort only) was considered useful to most staff, although one reported a concern about the burden of having more equipment to keep clean at the bedside.

S5: ‘The central monitoring screen gives a better view of the observations, and you can see straight away what the patient is triggering for’.

Staff reported that they value being able to tailor the silencing of the AMS alerts. This was seen by most as an improvement over the Philips monitor's three‐minute silence function, as there was time to address known issues or wait for specialist reviews. However, one nurse did feel that longer than 3 min was inappropriate for an unstable patient.

S2: ‘Five minutes is too long for a poorly patient, so three minutes is fine. You cancel it, and then it will ping again. After three minutes, I can check the patient’.

In response to increasing societal concerns about data privacy, the topic guide included a question about perceptions of data use. Patients reported that they were not worried about data security, trusting that safeguards were in place. In particular, they viewed their vital signs data as not particularly sensitive and that they were comfortable that displaying them prominently on the ward served a purpose in terms of their safety. No patient participant voiced concern about non‐clinical staff on the ward being able to see their vital signs.

P6: ‘As far as I'm concerned, I trust you. The people who are looking at my information are those caring for me, and the more people that have access to it, the better’.

Staff found the WMS intuitive and effective, with clear alerts and easy access to real‐time data. Some suggested more flexibility to tailor alarm settings according to clinical need (e.g., such further enhancing personalised care). Patients trusted the system's data security and felt unconcerned about who accessed their vital signs, viewing the information as non‐sensitive.

## 8 Discussion

This study investigated user experiences of a novel WMS implemented on a surgical ward as part of an RCT. Nurses, clinical support workers and patients described the WMS as giving them a sense of safety, supporting quick recognition and management of clinical deterioration. Nurses described the system as supporting workload management, but both patients and staff emphasised the importance of WMS not replacing nurse–patient contact. There was consensus that the unique WMS was acceptable for use in clinical practice.

This study builds on previous qualitative evaluations of WMS, which have considered the nurse (Kooij et al. 2022; van Zeist‐de Jonge et al. 2025), patient (Downey et al. 2018) and combined (van Noort et al. 2024) views of implementing WMS in clinical practice. Whilst both nurses and patients in this study agreed with the findings of previous studies that WMS support patient safety and rapid detection of deterioration (Buss et al. 2024; Kooij et al. 2022; Leenen et al. 2022) (also shown in small‐scale observational studies and feasibility trials (Aaberg et al. 2021; Areia, Biggs, et al. 2021)), this study further develops our understanding of user experiences of wearable monitoring. We explored the impact of these on nursing workflow from both staff and patient perspectives. Despite being implemented into nursing practice, this has not been as widely considered as the impact of WMS on patient outcomes. In a previous study, nurses reported that the system supported workload management and task prioritisation under highly pressured conditions during the COVID‐19 pandemic (Buss et al. 2024), whilst in an earlier study, nurses reported uncertainty about how the WMS they were using would fit into clinical workflows (Leenen et al. 2022). In a recent study, surveyed patients also perceived WMS to support nursing workload (Joshi et al. 2021). In this study, in the absence of the pressure induced by pandemic conditions, both patients and staff still reported that the WMS supported nurse workload by aiding prioritisation of tasks, as well as having the potential to decrease the time taken to measure vital signs.

In addition to improving patient safety and nurse workload, in this study, patients also identified benefits to their well‐being, which supported recovery. They valued the ability to view their own vital signs, offering reassurance, as well as facilitating faster treatment adjustments (e.g., oxygen therapy), supporting an individualised care plan tailored to the patient's recovery and improved mobility and autonomy through wireless monitoring. They also expressed a preference for fewer disturbances during sleep, which a central monitoring and alerting system could potentially address.

Despite the perceived benefits to patient safety and recovery, nurse workload and in common with earlier studies (Areia, Biggs, et al. 2021; Buss et al. 2024; Downey et al. 2018; van Zeist‐de Jonge et al. 2025) both patients and nurses emphasised the need for WMS not to replace nurse–patient contact. Therefore, a balance should be sought between using the system to support patient safety and nurse workload, and ensuring the important contact with nurses continues. This was clearly articulated by participants in this study, with nurses suggesting that the system could support nurse workload by utilising the available data rather than duplicating measurement with ward equipment.

Previous studies have highlighted concerns from both patients and staff about wearability, false alerts and poor usability of WMS (Downey et al. 2018; Kooij et al. 2022; Leenen et al. 2022; Weenk et al. 2020). Development of the WMS used in this study was informed by wearability, accuracy and acceptability testing (Areia et al. 2020; Areia, Biggs, et al. 2021; Santos et al. 2022) and the user interface was co‐developed with clinical staff on the ward. The system was then implemented into clinical practice during the pandemic (Buss et al. 2024; Santos et al. 2021) and then refinements were made based on nurse feedback. Perhaps because of this careful development, patients in this study found the WMS generally acceptable and nurses described the system as highly usable. In particular, the alerting system was optimised to reduce false alerts based on poor signal, movement artefact and poor connectivity (noted in previous studies as essential to successful deployment (Areia et al. 2022; Areia, Biggs, et al. 2021; Leenen et al. 2022)) and nurses had the facility to silence alerts remotely at the nurses' station. However, in agreement with earlier studies, both nurses and patients suggested that the WMS was only beneficial to those in the early stages of their illness and should be removed once patients were nearing discharge from the hospital. In particular, the pulse oximeter became more cumbersome as patients recovered. These findings underscore the need for ongoing refinement of wearable technologies to optimise comfort, accuracy and practicality in clinical settings, and clear protocols for use.

### 8.1 Strengths and Limitations of the Work

Patient outcomes related to health information technology are influenced by nurses' ability to act upon the new data it provides (Jeskey et al. 2011), as well as the extent to which patients can comply with the intervention. A key strength of this study was enabling both staff and patients to share their experiences together, offering valuable insights into their contrasting perspectives on the advantages and limitations of the system.

The research team members who conducted the interviews were known by the participants and had experience working in a ward environment, which, in the case of the staff interviews, facilitated in‐depth discussions that included professional language and context. An iterative process was followed, and the adequacy of the sample was confirmed as a team when data saturation was achieved (i.e., no new codes were generated from the final two interviews). Several steps were taken to maximise trustworthiness and alleviate researcher bias due to participant familiarity, including reflexive memo writing and regular team discussions throughout analysis.

Patient participants had some issues with recall, with some participants having vague memories of the periods when they were most unwell. Several patients required prompting to recall the devices they wore, which may reflect their unobtrusiveness.

Despite these strengths, there were some limitations that should be considered. The single‐site, single‐ward setting limits the broader transferability of findings to different settings and populations. Patient interviews were conducted either during their hospital stay or soon after hospital discharge, depending on appropriateness and participant preference. It is possible that the quality of data could have been impacted by memory recall in those who were interviewed after discharge. In addition, the different settings (in person, via Teams or telephone) may have influenced responses. Conducting interviews remotely may impair rapport with participants, which is important to ensure the interview is open and honest. However, all participants provided informed consent in person prior to the interview, so they had the opportunity to meet the researcher, and extra time was allocated at the start of remote interviews to ensure the participant was comfortable with the researcher. The potential impact of variation in timings and settings was recorded in the reflexive section of the CRF and discussed during analysis conversations. Although saturation was achieved overall, we were only able to interview two CSWs. As CSWs have an important and different role from nurses in vital signs monitoring, the recruitment of further CSWs may have provided additional insights. Additionally, staff interviews focused on those who used the system most in their practice, but the perspectives of physiotherapists and medical staff may have added additional dimensions to the data. Future research could investigate wider staff experiences and views. Whilst the sample size was small, it was sufficient for the relatively narrow focus and limitation to nursing, clinical support worker and patient views (Malterud et al. 2016). Additionally, the eligibility criteria of the feasibility RCT required participants to be able to understand English. This meant that no non‐English speakers were eligible for participation in the interview study. This is a limitation that should be addressed as part of a larger, multi‐centre study that includes diverse patient groups and includes language translation and interpretation services.

### 8.2 Recommendations for Further Research

In the associated feasibility RCT, routine observation rounds were conducted as usual alongside continuous monitoring. However, our findings suggest that both staff and patients would prefer an integrated approach that preserves normal staff‐patient interactions whilst reducing interruptions for vital signs measurement, particularly during times of sleep or rest. Further research is needed to understand the impact of WMS on staff‐patient dynamics and to develop evidence‐based guidelines that optimise the balance between automation and personal care. Additionally, further research could enhance alarm systems, including the development of patient‐specific alert thresholds, to ensure prompt responses to deterioration without generating excessive false positives, which contribute to alarm fatigue.

Whilst significant progress has been made in wearable health technologies, further innovation is needed to create wireless, wearable devices that reliably monitor all vital signs with hospital‐grade accuracy and are comfortable for patients of all ages, fragilities and stages of ill health.

In this study, the interviewed patients were generally in stable conditions. Nurses, however, emphasised that the monitoring system's value likely increases with the severity of patient illness. Therefore, future research should focus on identifying patient populations who would most benefit from wearable monitoring.

### 8.3 Implications for Policy and Practice

The recently published NHS Ten Year Plan highlights the need for healthcare to move from analogue to digital (Government U 2025), whilst the Darzi report into NHS services recommended a ‘tilt towards technology’ to support healthcare staff efficiency (Darzi 2024). The use of WMS has the potential to fulfil these urgent requirements for healthcare by using technology to improve patient safety and staff efficiency whilst caring for patients at risk of deterioration. However, careful consideration must be given to how this is used, to avoid over‐medicalisation and resource inefficiency if used in more stable patient groups. Policies should focus on clear implementation frameworks; robust staff training, including how central alerting systems require team oversight and action; and improving alarm specificity to reduce false positives and enhance response efficiency. Investment in more accurate, wearable devices and multi‐centre evaluations is needed to support broader adoption and scalability in diverse healthcare settings.

## 9 Conclusion

This study shows that both patients and staff found wearable monitoring in a clinical ward setting acceptable. Wearable devices provided real‐time vital sign monitoring, which patients felt improved safety and recovery without impacting their comfort. Staff reported that these devices had the potential to improve work efficiency and foster multi‐disciplinary team working, thereby improving patient care. Both groups agreed that centralised monitoring should not replace face‐to‐face clinical contact, and careful consideration should be given to who would benefit most from the technology.

## Author Contributions

C.E., methodology; project administration; data curation; formal analysis; writing – original draft; writing – review and editing. H.E., formal analysis; writing – original draft; writing – review and editing. E.K., formal analysis; writing – original draft; writing – review and editing. C.R., software design; writing – review and editing. P.W., conceptualisation; funding acquisition; methodology. S.V., conceptualisation; methodology; supervision; project administration; formal analysis; writing – review and editing. S.V., and C.E., conducted interviews. C.E., and H.E., coded and analysed the staff interviews. E.K., coded and analysed patient interviews. S.V., C.E., H.E., and E.K., developed the analysis. C.E., and H.E., drafted the first manuscript with all authors reviewing and finalising the first version for submission.

## Funding

This research was funded by the National Institute for Health Research (NIHR) Oxford Biomedical Research Centre (BRC) (NIHR203311). The views expressed are those of the author(s) and not necessarily those of the NHS, the NIHR or the Department of Health.

## Conflicts of Interest

The authors declare no conflicts of interest.

## Supporting information


**Data S1:** jan70610‐sup‐0001‐DataS1.docx.

## Data Availability

The data that support the findings of this study are available from the corresponding author upon reasonable request.
