# Supplementary material for: You Can't Be With Your Patients All the Time—Patient and Staff Views of a Wearable Vital Signs Monitoring System
Source: J Adv Nurs. 2026 Apr 20;82(11):9907–17. doi: 10.1111/jan.70610 (PMC13661789; doi:10.1111/jan.70610)
Supplement: Supplementary file 1 [file JAN-82-9907-s001.docx]

**Supplementary Material: Topic Guides**

**PATIENT INTERVIEW SCHEDULE**

**Questions and prompts**

1. Could you start by telling me what you understand by the term ‘monitoring’?

- *Why were you monitored?*
- *How did it feel to be monitored?*
- *Was this the first time you had been monitored?*
- *Did you go to ICU?*

2. During your admission, a bedside monitor may have continuously monitored you with wires

attached to your chest and hand.

- *What was good about this and what was bad about this?*
- *Were you ever the only patient in your bay who had a continuous monitor on and how did that feel?*
- *Did you experience any alarms from these systems? Tell me a little bit about that?*
- *In the context of your other invasive lines (drip or drain), how did you feel about the wired monitoring?*

3. During your admission you also wore a portable monitor for our study.

- *What was good about this and what was bad about this?*
- *Were you ever the only patient in your bay who had a continuous monitor on and how did that feel?*
- *Did you experience any alarms from these systems? Tell me a little bit about that?*
- *In the context of your other invasive lines (drip or drain), how did you feel about the wired monitoring?*

4. (If monitored by both systems): What was different about being monitored by these two systems?
5. Did you like being monitored?

- *What was good about it?*
- *What was bad about it?*

6. Do you think anything could improve patient monitoring?

**STAFF INTERVIEW SCHEDULE**

**Questions and prompts**

1. Can you talk to me about how you used the monitoring system for patients on your ward?

- *Which patients was it useful for?*
- *Which patients wasn’t it useful for?*
- *Would you have liked to use it on all your patients?*

2. What problems were there with the system?

- Hardware (patient wearability/ease of use)?
- Software (how you used the system)?
- Viewing the data?
- Are there any missing functionalities?

3. What were the benefits of the system?

4. Did you feel confident in the information the system gave you?

- Are there any additional vital signs you would like to monitor with this system?

5. How did you use the continuous data alongside usual observations?

- *Did you record/review partial observations sets on SEND?*
- *How did you use the central station alongside SEND?*
- *Would you like the waveforms on the dashboard?*
- *Do you think SEND and vHDU should be merged into one interface?*

6. Can you talk to me about how you used the alerting system

- *Was it useful?*
- *What could be changed to improve it?*
- *Did you trust it?*

7. What could be improved?

8. Would you like to continue using it?
   - *Why/why not?*
